# Development of short‐target primers for species identification in biological studies of Carnivora

**DOI:** 10.1002/ece3.10135

**Published:** 2023-05-25

**Authors:** Huiwen Liu, Dan Wang, Chenglin Zhang, Tianchun Pu, Lijuan Xiong, Fuwen Wei, Yibo Hu

**Affiliations:** ^1^ CAS Key Laboratory of Animal Ecology and Conservation Biology Institute of Zoology, Chinese Academy of Sciences Beijing China; ^2^ University of Chinese Academy of Sciences Beijing China; ^3^ Beijing Zoo Beijing China; ^4^ School of Life Sciences Guizhou Normal University Guiyang China

**Keywords:** Carnivora, COI, noninvasive sample, species identification

## Abstract

Noninvasive genetic sampling greatly facilitates studies on the genetics, ecology, and conservation of threatened species. Species identification is often a prerequisite for noninvasive sampling‐based biological studies. Due to the low quantity and quality of genomic DNA from noninvasive samples, high‐performance short‐target PCR primers are necessary for DNA barcoding applications. The order Carnivora is characterized by an elusive habit and threatened status. In this study, we developed three pairs of short‐target primers for identifying Carnivora species. The COI279 primer pair was suitable for samples with better DNA quality. The COI157a and COI157b primer pairs performed well for noninvasive samples and reduced the interference of nuclear mitochondrial pseudogenes (numts). COI157a could effectively identify samples from Felidae, Canidae, Viverridae, and Hyaenidae, while COI157b could be applied to samples from Ursidae, Ailuridae, Mustelidae, Procyonidae, and Herpestidae. These short‐target primers will facilitate noninvasive biological studies and efforts to conserve Carnivora species.

## 1 INTRODUCTION

Noninvasive sampling approaches are rapidly becoming the main method of collecting samples to study and conserve threatened species (Kelly et al., 2012), and for some large, threatened animals, this method is the most feasible solution (Laguardia et al., 2015; Schmidt et al., 2017). In this method, noninvasive samples (e.g., scats, hair, feathers, historical specimens) are collected without catching or killing the animals, which causes minimal disturbance to them (Taberlet et al., 1999). Researchers can extract DNA from these noninvasive samples to gain a variety of information, such as species identification, individual identification, sex, diet composition, and gut microbiome (Chaves et al., 2012; DeCandia et al., 2016; Rodgers & Janečka, 2013). In addition, species identification is also a prerequisite for other studies of wild animals, including behavioral (e.g., sexual selection), physiological (e.g., scat hormone measure), and animal medicine (e.g., parasite infection rate) studies (Huang et al., 2014; Schwartz & Monfort, 2008; Wei et al., 2012).

Carnivora, as the fourth largest order of Mammalia, harbors many top predators and threatened species (Yu & Zhang, 2006), which receive wide public attention and conservation efforts worldwide. Due to the low population size, high vigilance and elusiveness, and often nocturnal habit of these species, it is difficult to directly observe wild carnivores, which greatly hinders field surveys and conservation biology research. Under these circumstances, noninvasive sampling has become an appropriate and widely used solution for the study and conservation of wild carnivores (Zemanova, 2021), especially Felidae, Canidae, Ursidae, and Musteloidea (e.g., Bischof et al., 2016; Modi et al., 2018; Mumma et al., 2015; Wang et al., 2016).

DNA barcoding is a widely used method of molecular species identification in species diversity and conservation genetics studies. Hebert et al. (2003) first proposed the use of the mitochondrial cytochrome C oxidase subunit I (*COI*) gene fragment as a marker for DNA barcoding. For species identification with Carnivora animals, widely used universal *COI* primers (e.g., primers developed for metazoans, vertebrates, or mammals) were used in most studies, such as LCO1490/HCO2198 (710‐bp product length) (Folmer et al., 1994), VF1/VR1, VF1d/VR1d, VF1i/VR1i (658‐bp product length) (Ivanova et al., 2006; Ward et al., 2005), GF_1/GR_1 (approximately 650‐bp product length) (Li et al., 2017), and COI_minimm_F/COI_minimm_R (102‐bp product length) (Kocher et al., 2017). Only one study designed specific *COI* primers, BC‐F2/BC‐F3/BC‐R2 (239‐bp or 188‐bp product length), for Carnivora species (Chaves et al., 2012).

The application of universal primers developed for vertebrates or mammals in Carnivora could pose some potential problems. First, these primers are often tested in a limited number of species, which may limit their utility in some particular groups. Second, interference from nuclear mitochondrial pseudogenes (numts) exists in some groups of Carnivora. For example, numts of *COI* have been reported in *Panthera* (Kim et al., 2006), *Felis* (Lopez et al., 1994), and *Otocolobus* (Pietsch, 2012). These universal primers might amplify numts, which would introduce errors in species identification and phylogenetic analysis (Zhang & Hewitt, 1996). Third, because DNA that is extracted from noninvasive samples is often degraded, some universal primers with relatively long product lengths might fail to amplify efficiently (Taberlet et al., 1999). Therefore, it is necessary and practical to design universal primers not only for identifying Carnivora species but also for noninvasive samples.

In this study, we designed three pairs of universal Carnivora primers using the consensus‐degenerate hybrid oligonucleotide primer (CODEHOP) method (Boyce et al., 2009; Rose et al., 1998, 2003) and manual alignment methods and evaluated the performance of these primers using Carnivora samples from 38 species and three subspecies, which consisted of 33 tissue samples from 31 species and 20 scat samples from 19 species.

## 2 MATERIALS AND METHODS

### 2.1 Sample collection and DNA extraction

Tissue samples of dead individuals from 31 Carnivora species in nine families (Canidae, Ursidae, Ailuridae, Mephitidae, Mustelidae, Procyonidae Felidae, Viverridae, and Herpestidae) and scat samples from 19 Carnivora species in six families (Canidae, Ursidae, Ailuridae, Procyonidae, Felidae, and Hyaenidae) were collected with specific sources shown in Table A1. These samples were stored in −80°C freezers. The collections of tissue samples of dead animals and scat samples were approved by the agencies that the samples were affiliated with (Table A1) and conform to the guidelines of animal care and experiments. Tissue DNA was extracted with a DNeasy Blood & Tissue Kit (QIAGEN), and scat DNA was extracted with a QIAamp Fast DNA Stool Mini Kit (QIAGEN) following the manufacturer's instructions. After extraction, DNA was visualized in 1% agarose gels to assess its quality by observing whether the electrophoresis band was clear. Because of the limited scat volume and multiple PCR experiments, some scat samples were not used for all three pairs of primers.

### 2.2 Primer design

The CODEHOP strategy can be used to design universal primers to perform PCR amplification with distantly related gene sequences. Designed from conserved amino acids within aligned protein sequences, a CODEHOP primer consists of a 3′ degenerate core region and a 5′ nondegenerate ‘clamp’ region. The 3′ core region, with a length of approximately three to four amino acids, is in a highly conserved area. To amplify more potential sequences, the 3′ core region uses a degenerate base in the third position of each codon. The 5′ clamp region, which has a length of approximately five to seven amino acids, uses the most common nucleotide of reference sequences. It does not contain degenerate bases, which ensures amplification stability and efficiency during the annealing process of the later cycles (Boyce et al., 2009; Rose et al., 1998, 2003). Reference *COI* sequences of 126 Carnivora species were obtained from NCBI GenBank (Table A2). Conserved regions of the *COI* gene were preliminarily screened in ClustalX (version 2.1; Larkin et al., 2007) by sequence alignment, and the *COI* codon usage bias of carnivores was calculated by CodonW (version 1.4.2; http://codonw.sourceforge.net/).

**FIGURE 1:**
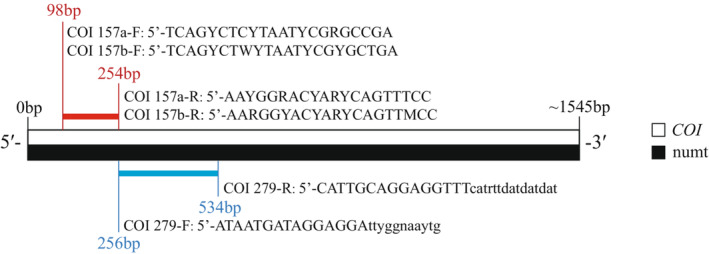
Specific locations of universal Carnivora primers (COI 157a/b and COI 279) designed on the *COI* gene sequence. The whole *COI* gene is located within the numt regions of *Felis* or *Panthera* genera (Kim et al., 2006; Lopez et al., 1994).

Reference *COI* sequences and codon usage bias were imported to Base‐By‐Base (version 3; Tu et al., 2018), a software package including the j‐CODEHOP plug‐in. The max degeneracy parameter was set to 32 to find as many potential universal Carnivora primers as possible, while clamp length was set to 15 bp and core length to four amino acids to gain primers of suitable length. Then, selected primers were evaluated by the online calculator OligoCalc (2021‐3‐14; Kibbe, 2007). In this step, primers were matched according to melting temperature. The poly‐G/C sequence, potential hairpin formation, self‐annealing, and cross‐dimer were checked to filter out improper primers. A universal Carnivora primer pair COI279 (F: 5′‐ATAATGATAGGAGGAttyggnaaytg; R: 5′‐CATTGCAGGAGGTTTcatrttdatdatdat) was selected, in which the uppercase section is for the clamp region and the lowercase section is for the core region. The product length was 279 bp (Figure 1), a length that could be amplified in noninvasive samples (e.g., Huber et al., 2002).

Because numts are present in Felidae as reported previously (Kim et al., 2006; Lopez et al., 1994; Pietsch, 2012), we manually designed two shorter universal Carnivora primer pairs to avoid the impacts of numts. Based on the sequence alignment results, we selected the sequences with high conservativeness among felids but multiple variations when comparing with numts, as the potential primers. Then, we used Primer Premier 5 (version 5.00; Lalitha, 2000) to assess the quality of the primer pairs based on the occurrence of hairpins, self‐annealing, and cross dimers. COI157a (F: 5′‐TCAGYCTCYTAATYCGRGCCGA; R: 5′‐AAYGGRACYARYCAGTTTCC) was designed for identifying the Canidae, Felidae, and Viverridae samples, while COI157b (F: 5′‐TCAGYCTWYTAATYCGYGCTGA; R: 5′‐AARGGYACYARYCAGTTMCC) was designed for identifying the samples from the other Carnivora families. The two primer pairs produced 157‐bp PCR products (Figure 1), which was a suitable length for amplifying seriously degraded noninvasive samples (Michalski et al., 2011; Santini et al., 2007).

### 2.3 PCR amplification

For the amplification of COI279, COI157a, and COI157b primers, a 30‐μL PCR system was used, including 15 μL of HotStar‐Taq Master Mix (QIAGEN), 0.1 μg/μL BSA (TaKaRa), 0.25–0.5 μM of each COI279 primer or 0.25–0.33 μM of each COI157a/157b primer, and 3–5 μL of DNA template (depending on the PCR amplification performance). For COI279, the amplification procedure consisted of an initial denaturation (15 min at 95°C) followed by touchdown PCR (10 cycles of 30 s at a denaturation temperature of 94°C; 40 s at an annealing temperature of 62–54°C, which was decreased by 2°C every 2 cycles; and 50 s at an extension temperature of 72°C), followed by 30–35 cycles (depending on the DNA quality) of 30 s at 94°C, 40 s at 52°C, and 50 s at 72°C, and a final extension for 10 min at 72°C. For COI157a and COI157b, the amplification procedure consisted of an initial denaturation (15 min at 95°C) followed by touchdown PCR (a total of eight cycles of 30 s at 94°C; 40 s at a temperature from 54 to 52°C, which was decreased by 2°C every four cycles; and 50 s at 72°C), followed by 30–35 cycles of 30 s at 94°C, 40 s at 50°C, and 50 s at 72°C, and a final extension for 10 min at 72°C. All PCR products were visualized in 2% agarose gels. Then, the positive products were recovered separately by Gel DNA Recovery Cleaning Kit (GeneOn Biotech) and cleaned by BigDye Sequencing Clean Up Kit (MCLAB). Finally, the products were sequenced bidirectionally on an ABI 3730XL Analyzer (Thermo Fisher, at SinoGenoMax company) to obtain reliable results.

### 2.4 Species identification

Bidirectional DNA sequences were assembled by the SeqMan module of DNAStar software (version 7.1.0; Swindell & Plasterer, 1997). Only clear sequence peaks were considered credible. Next, sequence searches were executed by megaBLAST of NCBI to identify the species source of the samples, and the results were then compared with the known species source of the samples.

## 3 RESULTS

All three pairs of universal Carnivora primers worked effectively for the Carnivora species samples. The COI279 primer pair performed well for the samples of 26 species from nine families and 23 genera of Carnivora (Table 1). However, this primer pair also had limitations. The first problem was interference from numts. Although the samples of *Felis* and *Otocolobus* could be identified successfully, the samples of *Panthera*, such as leopard and snow leopard samples, could not be identified because of interference from numts, and the chromatograms were disordered as a result. Furthermore, BLAST sequences for the *Vulpes* species often revealed *Nyctereutes procyonoides* with a similarity of 86%; thus, COI279 cannot be used to recognize foxes. For noninvasive samples, COI279 identified four of seven scat samples tested (Table 1, Table A5).

**TABLE 1:** Species identification results using COI279 and COI157a/b primers designed in this study.

Family	Species or subspecies	English name	PCR identification	
Canidae	*Canis lupus familiaris*	Dog	COI279	COI157a
	*Canis lupus*	Gray wolf	N/A	**COI157a**
	*Canis mesomelas*	Black‐backed jackal	N/A	**COI157a**
	*Cuon alpinus*	Dhole	COI279	COI157a
	*Nyctereutes procyonoides*	Raccoon dog	COI279	**COI157a**
	*Vulpes corsac*	Corsac fox	N/A	Failed
	*Vulpes lagopus*	Arctic fox	Failed	Failed
	*Vulpes vulpes*	Red fox	Failed	**COI157a**
	*Vulpes ferrilata*	Tibetan fox	Failed	Failed
Felidae	*Felis catus*	Cat	COI279	COI157a
	*Acinonyx jubatus*	Cheetah	N/A	**COI157a**
	*Caracal caracal*	Caracal cat	COI279	Failed
	*Lynx lynx*	Eurasian lynx	COI279	**COI157a**
	*Otocolobus manul*	Pallas's cat	COI279	**COI157a**
	*Puma concolor*	Puma	COI279	COI157a
	*Panthera leo*	Lion	Failed	**COI157a**
	*Panthera tigris tigris*	Bengal tiger	COI279	**COI157a**
	*Panthera tigris altaica*	Amur tiger	COI279	**COI157a**
	*Panthera pardus orientalis*	Amur leopard	Failed	COI157a
	*Panthera pardus delacouri*	Indochinese leopard	N/A	**COI157a**
	*Panthera onca*	Jaguar	Failed	**COI157a**
	*Panthera uncia*	Snow leopard	Failed	COI157a
	*Prionailurus bengalensis*	Leopard cat	COI279	COI157a
Viverridae	*Paguma larvata*	Masked palm civet	COI279	COI157a
Hyaenidae	*Hyaena hyaena*	Striped hyaena	Failed	**COI157a**
Herpestidae	*Suricata suricatta*	Meerkat	COI279	COI157b
Mephitidae	*Mephitis mephitis*	Striped skunk	COI279	Failed
Ailuridae	*Ailurus styani*	Chinese red panda	**COI279**	**COI157b**
Mustelidae	*Lutra lutra*	Eurasian otter	COI279	COI157b
	*Martes flavigula*	Yellow‐throated marten	COI279	COI157b
	*Meles meles*	Badger	COI279	COI157b
	*Arctonyx collaris*	Hog‐badger	COI279	COI157b
	*Melogale moschata*	Chinese ferret‐badger	COI279	COI157b
	*Mustela putorius furo*	Ferret	COI279	COI157b
	*Mustela sibirica*	Siberian weasel	COI279	COI157b
Procyonidae	*Procyon lotor*	Northern raccoon	N/A	**COI157b**
	*Potos flavus*	Kinkajou	**COI279**	**COI157b**
Ursidae	*Ursus maritimus*	Polar bear	COI279	COI157b
	*Ursus arctos*	Brown bear	**COI279**	**COI157b**
	*Ursus thibetanus*	Asiatic black bear	COI279	COI157b
	*Ailuropoda melanoleuca*	Giant panda	**COI279**	COI157b

*Note*: Regular without bold represents that tissue samples were successfully identified; bold means that scat samples were successfully identified; bold with underline means that both tissue and scat samples were successfully identified; N/A means that the sample was missing or exhausted; “Failed” represents the failures of PCR amplification, Sanger sequencing, or species identification.

The COI157a primer pair successfully identified most samples (18 of 22 species tested) from Felidae, Canidae, Viverridae, and Hyaenidae (Table 1). It can successfully overcome the interference from numts for *Panthera* samples. This primer pair had difficulties distinguishing related *Vulpe*s species, such as *Vulpes corsac* and *V. ferrilata* as well as *V. lagopus* and *V. velox*. It cannot discriminate *Caracal caracal* from *Prionailurus rubiginosus*. For noninvasive samples, the COI157a primer pair successfully identified scat samples from 13 of 15 species tested from three families (Table A3).

The COI157b primer pair successfully identified most samples (15 of 16 species tested) from Ailuridae, Ursidae, Herpestidae, Mustelidae, and Procyonidae (Table 1). However, it failed to identify *Mephitis mephitis* because the PCR was unsuccessful. For noninvasive samples, the COI157b primer pair successfully identified scat samples from four of five species tested from three families (Table A4).

## 4 DISCUSSION

In this study, the universal Carnivora primers COI157a and COI157b performed well for most of the tested species, which represented multiple different families, and for the amplification of noninvasive samples. For most Carnivora species, species identification can be performed by combining two pairs of universal Carnivora primers. Compared to the other primers, COI279 showed higher universality for many Carnivora species, although we lacked enough samples to test whether COI279 is suitable for pinnipeds.

Nevertheless, COI279 had some limitations. First, because the product length was 279 bp, COI279 was a better option for good‐quality DNA samples. The second limitation is interference from numts. In addition to being unable to identify *Panthera* species, COI279 also amplified unknown sequences from *Vulpes* species. Because the unknown sequences BLASTed to the *Vulpes vulpes* genome, we speculate that it may be the newly discovered numt of the *Vulpes*. This is possible because a nearly complete mitochondrial genome was discovered in the nuclear genome of dogs (*Canis lupus familiaris*) (Verscheure et al., 2015), implying the possible existence of unknown numts in other Canidae species.

There is no doubt that numt interference on universal primers is a large obstacle for the popularization and application of the DNA barcoding method. Coamplification could occur easily. However, for ancient and conserved numts relative to homologous mtDNA, universal primers may tend to amplify numts rather than mtDNA (Bensasson et al., 2001). For taxonomical groups with known numts, numts could be avoided by manually designing primers. For taxonomical groups in which the existence of numts is unknown, caution is needed when analyzing the mtDNA data and especially when assembling the mitochondrial genome as a reference for DNA barcoding. Alternative methods of reducing numt interference include extracting mitochondrial DNA to reduce the impacts of numts (Ibarguchi et al., 2006) and recognizing numts by checking for the occurrence of indels, in‐frame stop codons, and changes in nucleotide composition in the sequences (Song et al., 2008). Recent studies have suggested that bioinformatics filtering of high‐throughput sequencing data may be a good choice for discovering unknown numts (Smart et al., 2019; Woerner et al., 2020).

For noninvasive samples, we designed COI157a and COI157b universal Carnivora primers with short PCR products, which were also named DNA minibarcoding (Meusnier et al., 2008). In our study, the universal Carnivora primers COI157a and COI157b performed well for noninvasive samples. With the rapid development of next‐generation sequencing technology, the two primer pairs could be used as potential molecular markers for high‐throughput species identification. However, in our study, short products had difficulties in differentiating subspecies or most closely related species, that is, *Canis lupus* and *C. l. familiaris*, *Felis catus* and *F. silvestris*, *Panthera tigris tigris* and *P. t. altaica*, because short products have less variation for subspecies or most closely related species.

There were some troubles in PCR experiments of noninvasive samples under some circumstances. In this study, we used touchdown PCR (Korbie & Mattick, 2008) to increase the specificity and sensitivity of PCR. Considering the presence of PCR inhibitors in noninvasive DNA extracts, especially scat samples, we added BSA to the PCR solution. Diluting DNA extracts or reducing the amount of DNA extracts used are also optional solutions (Schrader et al., 2012). In addition, it has been reported that scat samples may coamplify both predator DNA and prey DNA (Symondson, 2002). We did not find the signature of prey DNA sequences in our BLAST identification; however, this remains a problem that should be treated with caution in scat DNA studies.

Our universal Carnivora primers for species identification have some advantages over existing similar primers. For COI primers, LCO1490/HCO2198 (Folmer et al., 1994) is a widely used primer pair in Carnivora species identification. However, its 710‐bp product is not suitable for noninvasive samples. Compared with our primers, the BC‐F2/BC‐F3/BC‐R2 primer (Chaves et al., 2012), which is for Carnivora species identification, has a longer product and was tested on fewer families and species of Carnivora. Additionally, compared with other non‐COI Carnivora noninvasive primers (De Barba et al., 2014; Michalski et al., 2011; Rodriguez‐Castro et al., 2020), our primers also have some advantages: for example, using only one pair of primers, obtaining DNA sequences rather than the fragment length, or having wider universality to Carnivora species.

In summary, our developed short‐fragment primers could have important applications for species identification in noninvasive studies of Carnivora species, especially in conservation genetics, individual‐based ecology, and metagenomics studies.

## AUTHOR CONTRIBUTIONS

**Huiwen Liu:** Formal analysis (lead); writing – original draft (lead). **Dan Wang:** Formal analysis (lead). **Chenglin Zhang:** Resources (equal). **Tianchun Pu:** Resources (equal). **Lijuan Xiong:** Formal analysis (supporting). **Fuwen Wei:** Funding acquisition (equal); resources (equal); writing – review and editing (supporting). **Yibo Hu:** Conceptualization (lead); funding acquisition (equal); project administration (lead); supervision (lead); writing – review and editing (lead).

## CONFLICT OF INTEREST STATEMENT

The authors declare no conflicts of interest.

## Data Availability

The COI DNA sequences of Carnivora species obtained in this study have already existed in the NCBI database, and no sequences from new Carnivora species that previously did not have COI sequences in the NCBI were reported.

## APPENDIX A

**TABLE A1:** Sources of the samples used in this study.

Family	Species or subspecies	English name	Type	Origin
Canidae	*Canis lupus familiaris*	Dog	T	Domestic
	*Canis lupus*	Gray Wolf	T, S	Zoo
	*Canis mesomelas*	Black‐backed Jackal	S	Zoo
	*Cuon alpinus*	Dhole	T	Zoo
	*Nyctereutes procyonoides*	Raccoon Dog	T, S	Wild (Shaanxi Province)
	*Vulpes corsac*	Corsac Fox	S	Zoo
	*Vulpes lagopus*	Arctic Fox	T	Zoo
	*Vulpes vulpes*	Red Fox	S	Zoo
	*Vulpes ferrilata*	Tibetan Fox	T	Wild (Qinghai Province)
Ursidae	*Ursus maritimus*	Polar Bear	T	Zoo
	*Ursus arctos*	Brown Bear	S	Zoo
	*Ursus thibetanus*	Asiatic Black Bear	T	Wild (Shaanxi)
	*Ailuropoda melanoleuca*	Giant Panda	T, S	Zoo
Ailuridae	*Ailurus styani*	Chinese Red Panda	T, S	Zoo
Mephitidae	*Mephitis mephitis*	Striped Skunk	T	Zoo
Mustelidae	*Lutra lutra*	Eurasian River Otter	T	Wild (Shaanxi)
	*Martes flavigula*	Yellow‐throated Marten	T	Wild (Shaanxi)
	*Meles meles*	Badger	T	Wild (Shaanxi)
	*Arctonyx collaris*	Hog‐Badger	T	Wild (Shaanxi)
	*Melogale moschata*	Chinese Ferret‐badger	T	Wild (Shaanxi)
	*Mustela putorius furo*	Ferret	T	Zoo
	*Mustela sibirica*	Siberian Weasel	T	Wild (Shaanxi)
Procyonidae	*Potos flavus*	Kinkajou	S	Zoo
	*Procyon lotor*	Northern Raccoon	T, S	Zoo
Felidae	*Felis catus*	Cat	T	Domestic
	*Acinonyx jubatus*	Cheetah	S	Zoo
	*Caracal caracal*	Caracal Cat	T, S	Zoo
	*Lynx lynx*	Eurasian Lynx	T, S	Zoo
	*Otocolobus manul*	Pallas's Cat	T, S	Zoo
	*Puma concolor*	Puma	T	Zoo
	*Panthera leo*	Lion	T, S	Zoo
	*Panthera tigris tigris*	Bengal Tiger	T, S	Zoo
	*Panthera tigris altaica*	Amur Tiger	T, S	Zoo
	*Panthera pardus orientalis*	Amur Leopard	T	Zoo
	*Panthera pardus delacouri*	Indo‐Chinese Leopard	S	Zoo
	*Panthera onca*	Jaguar	T, S	Zoo
	*Panthera uncia*	Snow Leopard	T	Wild (Qinghai)
	*Prionailurus bengalensis*	Leopard Cat	T	Zoo
Viverridae	*Paguma larvata*	Masked Palm Civet	T	Wild (Shaanxi)
Herpestidae	*Suricata suricatta*	Meerkat	T	Zoo
Hyaenidae	*Hyaena hyaena*	Striped Hyaena	S	Zoo

*Note*: T, tissue; S, scat.

**TABLE A2:** Sources of the sequences used in this study (NCBI GenBank accession number).

Family	Genus	Species	Accession number
Ailuridae	*Ailurus*	*Ailurus fulgens*	NC_011124.1
Canidae	*Canis*	*Canis aureus*	KT_448274.1
		*Canis lupus*	NC_010340.2
		*Canis latrans*	NC_008093.1
	*Cuon*	*Cuon alpinus*	NC_013445.1
	*Chrysocyon*	*Chrysocyon brachyurus*	NC_024172.1
	*Lycaon*	*Lycaon pictus*	NC_028427.1
	*Nyctereutes*	*Nyctereutes procyonoides*	NC_013700.1
	*Vulpes*	*Vulpes bengalensis*	NC_045533.1
		*Vulpes corsac*	NC_023958.1
		*Vulpes ferrilata*	NC_027935.1
		*Vulpes vulpes*	NC_008434.1
		*Vulpes lagopus*	NC_026529.1
		*Vulpes zerda*	NC_023459.1
	*Otocyon*	*Otocyon megalotis*	NC_036369.1
	*Urocyon*	*Urocyon cinereoargenteus*	NC_026723.1
Eupleridae	*Mungotictis*	*Mungotictis decemlineata*	NC_027828.1
Felidae	*Felis*	*Felis chaus*	NC_028307.1
		*Felis catus*	NC_001700.1
		*Felis nigripes*	NC_028309.1
		*Felis margarita*	NC_028308.1
		*Felis silvestris*	NC_028310.1
	*Caracal*	*Caracal caracal*	NC_028306.1
	*Catopuma*	*Catopuma temminckii*	NC_027115.1
		*Catopuma badia*	KX_265094.1
	*Profelis*	*Profelis aurata*	NC_028299.1
	*Acinonyx*	*Acinonyx jubatus*	NC_005212.1
	*Lynx*	*Lynx lynx*	NC_027083.1
		*Lynx canadensis*	NC_028313.1
		*Lynx pardinus*	KX_911412.1
		*Lynx rufus*	NC_014456.1
	*Leopardus*	*Leopardus jacobita*	NC_028322.1
		*Leopardus guigna*	NC_028321.1
		*Leopardus geoffroyi*	NC_028320.1
		*Leopardus wiedii*	NC_028318.1
		*Leopardus tigrinus*	NC_028317.1
		*Leopardus pardalis*	NC_028315.1
		*Leopardus colocolo*	NC_028314.1
	*Neofelis*	*Neofelis nebulosa*	NC_008450.1
	*Otocolobus*	*Otocolobus manul*	NC_028323.1
	*Puma*	*Puma concolor*	NC_016470.1
		*Puma yagouaroundi*	NC_028311.1
	*Panthera*	*Panthera pardus*	NC_010641.1
		*Panthera leo*	KX_258452.1
		*Panthera onca*	NC_022842.1
		*Panthera tigris*	NC_014770.1
		*Panthera uncia*	KP_202269.1
	*Leptailurus*	*Leptailurus serval*	NC_028316.1
	*Pardofelis*	*Pardofelis marmorat*	NC_028303.1
	*Prionailurus*	*Prionailurus bengalensis*	NC_016189.1
		*Prionailurus planiceps*	KY_682747.1
		*Prionailurus viverrinus*	NC_028305.1
Herpestidae	*Herpestes*	*Herpestes javanicus*	NC_006835.1
		*Herpestes ichneumon*	MW_019668.2
		*Herpestes brachyurus*	KY_117547.1
Hyaenidae	*Hyaena*	*Hyaena hyaena*	NC_020669.1
	*Crocuta*	*Crocuta crocuta*	NC_020670.1
	*Parahyaena*	*Parahyaena brunnea*	NC_038159.1
	*Proteles*	*Proteles cristata*	MH_662445.1
Mephitidae	*Conepatus*	*Conepatus chinga*	NC_042596.1
	*Mephitis*	*Mephitis mephitis*	NC_020648.1
	*Spilogale*	*Spilogale putorius*	NC_010497.1
Mustelidae	*Aonyx*	*Aonyx cinerea*	NC_035814.1
		*Aonyx capensis*	NC_046484.1
	*Arctonyx*	*Arctonyx collaris*	NC_020645.1
	*Gulo*	*Gulo gulo*	NC_009685.1
	*Taxidea*	*Taxidea taxus*	NC_020646.1
	*Lutra*	*Lutra lutra*	NC_011358.1
		*Lutra sumatrana*	NC_035810.1
	*Enhydra*	*Enhydra lutris*	NC_009692.1
	*Lutrogale*	*Lutrogale perspicillata*	NC_035811.1
	*Martes*	*Martes flavigula*	NC_012141.1
		*Martes martes*	NC_021749.1
		*Martes americana*	MT_028278.1
		*Martes foina*	NC_020643.1
		*Martes zibellina*	NC_011579.1
	*Meles*	*Meles leucurus*	NC_039173.1
		*Meles meles*	NC_011125.1
		*Meles anakuma*	NC_009677.1
	*Melogale*	*Melogale moschata*	NC_020644.1
	*Mustela*	*Mustela altaica*	NC_021751.1
		*Mustela erminea*	NC_025516.1
		*Mustela kathiah*	NC_023210.1
		*Mustela nivalis*	NC_020639.1
		*Mustela sibirica*	NC_020637.1
		*Mustela putorius*	KT_693383.1
		*Mustela frenata*	HM_106321.1
		*Mustela nigripes*	NC_024942.1
		*Mustela itatsi*	NC_034330.1
		*Mustela lutreola*	MT_304869.1
	*Vormela*	*Vormela peregusna*	MW_013133.1
Nandiniidae	*Nandinia*	*Nandinia binotata*	NC_024567.1
Odobenidae	*Odobenus*	*Odobenus rosmarus*	NC_004029.2
Otariidae	*Callorhinus*	*Callorhinus ursinus*	NC_008415.3
	*Arctocephalus*	*Arctocephalus forsteri*	KT_693378.1
	*Otaria*	*Otaria byronia*	NC_049152.1
	*Eumetopias*	*Eumetopias jubatus*	NC_004030.2
Phocidae	*Erignathus*	*Erignathus barbatus*	NC_008426.1

*Phoca*	*Phoca largha*	NC_008430.1

	*Phoca vitulina*	NC_001325.1

*Phoca groenlandica*	NC_008429.1

*Phoca fasciata*	NC_008428.1

*Pusa*	*Pusa hispida*	NC_008433.1

	*Pusa caspica*	NC_008431.1

*Pusa sibirica*	NC_008432.1

*Monachus*	*Monachus monachus*	NC_044972.1

*Lobodon*	*Lobodon carcinophaga*	NC_008423.1

*Hydrurga*	*Hydrurga leptonyx*	NC_008425.1

*Eptonychotes*	*Eptonychotes weddellii*	NC_008424.1

*Mirounga*	*Mirounga leonina*	NC_008422.1
Prionodontidae	*Prionodon*	*Prionodon pardicolor*	NC_024569.1
Procyonidae	*Procyon*	*Procyon lotor*	AM_711899.1
	*Nasua*	*Nasua nasua*	NC_020647.1
Ursidae	*Ailuropoda*	*Ailuropoda melanoleuca*	NC_009492.1
	*Helarctos*	*Helarctos malayanus*	NC_009968.1
	*Melursus*	*Melursus ursinus*	NC_009970.1
	*Tremarctos*	*Tremarctos ornatus*	NC_009969.1
	*Ursus*	*Ursus arctos*	NC_003427.1
		*Ursus thibetanus*	NC_009331.1
		*Ursus americanus*	NC_003426.1
		*Ursus maritimus*	AJ_428577.1
Viverridae	*Arctictis*	*Arctictis binturong*	NC_043895.1
	*Civettictis*	*Civettictis civetta*	NC_033378.1
	*Paguma*	*Paguma larvata*	NC_029403.1
	*Paradoxurus*	*Paradoxurus hermaphroditus*	NC_039591.1
	*Viverricula*	*Viverricula indica*	NC_025296.1

**TABLE A3:** Species identification results using COI157a primers.

Family	Genus	Species or subspecies	PCR	Sequencing	Type
Canidae	*Canis*	*Canis lupus familiaris*	Y	Y	T
		*Canis lupus*	Y	Y	T, S
		*Canis mesomelas*	Y	Y	S
	*Cuon*	*Cuon alpinus*	Y	Y	T
	*Nyctereutes*	*Nyctereutes procyonoides*	Y	Y	T, S
	*Vulpes*	*Vulpes corsac*	Y	N^1^	S
		*Vulpes vulpes fulva*	Y	Y	S
		*Vulpes lagopus*	Y	N^2^	T
		*Vulpes vulpes*	Y	Y	S
		*Vulpes ferrilata*	Y	N	T
Felidae	*Felis*	*Felis catus*	Y	Y	T
	*Acinonyx*	*Acinonyx jubatus*	Y	Y	S
	*Caracal*	*Caracal caracal*	Y	N	T, S
	*Lynx*	*Lynx lynx*	Y	Y	T, S
	*Otocolobus*	*Otocolobus manul*	Y	Y	T, S
	*Puma*	*Puma concolor*	Y	Y	T
	*Panthera*	*Panthera leo*	Y	Y	T, S
		*Panthera tigris tigris*	Y	Y	T, S
		*Panthera tigris altaica*	Y	Y	T, S
		*Panthera pardus orientalis*	Y	Y	T
		*Panthera pardus delacouri*	Y	Y	S
		*Panthera onca*	Y	Y	T, S
		*Panthera uncia*	Y	Y	T
	*Prionailurus*	*Prionailurus bengalensis*	Y	Y	T
Viverridae	*Paguma*	*Paguma larvata*	Y	Y	T
Hyaenidae	*Hyaena*	*Hyaena hyaena*	Y	Y	S

*Note*: Y, succeeded; N, failed; N^1^, unable to distinguish between *V. corsac* and *V. ferrilata*; N^2^, unable to distinguish between *V. lagopus* and *V. velox*; T, tissue; S, scat.

**TABLE A4:** Species identification results using COI157b primers.

Family	Genus	Species or subspecies	PCR	Sequencing	Type
Ursidae	*Ursus*	*Ursus maritimus*	Y	Y	T
		*Ursus arctos*	Y	Y	S
		*Ursus thibetanus*	Y	Y	T
	*Ailuropoda*	*Ailuropoda melanoleuca*	Y	Y^1^	T, S
Ailuridae	*Ailurus*	*Ailurus fulgens*	Y	Y	T, S
Mephitidae	*Mephitis*	*Mephitis mephitis*	N	N	T
Mustelidae	*Lutra*	*Lutra lutra*	Y	Y	T
	*Martes*	*Martes flavigula*	Y	Y	T
	*Meles*	*Meles meles*	Y	Y	T
	*Arctonyx*	*Arctonyx collaris*	Y	Y	T
	*Melogale*	*Melogale moschata*	Y	Y	T
	*Mustela*	*Mustela putorius furo*	Y	Y	T
		*Mustela sibirica*	Y	Y	T
Procyonidae	*Procyon*	*Procyon lotor*	Y	Y	T, S
		*Potos flavus*	Y	Y	S
Herpestidae	*Suricata*	*Suricata suricatta*	Y	Y	T

*Note*: Y, succeeded; N, failed; Y^1^, only succeeded in the tissue sample; T, tissue; S, scat.

**TABLE A5:** Species identification results using COI279 primers.

Family	Genus	Species or subspecies	PCR	Sequencing	Type
Canidae	*Canis*	*Canis lupus familiaris*	Y	Y	T
	*Cuon*	*Cuon alpinus*	Y	Y	T
	*Nyctereutes*	*Nyctereutes procyonoides*	Y	Y	T
	*Vulpes*	*Vulpes vulpes fulva*	Y	N	S
		*Vulpes lagopus*	Y	N	T
		*Vulpes vulpes*	Y	N	S
		*Vulpes ferrilata*	Y	N	T
Ursidae	*Ursus*	*Ursus maritimus*	Y	Y	T
		*Ursus arctos*	Y	Y	S
		*Ursus thibetanus*	Y	Y	T
	*Ailuropoda*	*Ailuropoda melanoleuca*	Y	Y	T, S
Ailuridae	*Ailurus*	*Ailurus fulgens*	Y	Y	T, S
Mephitidae	*Mephitis*	*Mephitis mephitis*	Y	Y	T
Mustelidae	*Lutra*	*Lutra lutra*	Y	Y	T
	*Martes*	*Martes flavigula*	Y	Y	T
	*Meles*	*Meles meles*	Y	Y	T
	*Arctonyx*	*Arctonyx collaris*	Y	Y	T
	*Melogale*	*Melogale moschata*	Y	Y	T
	*Mustela*	*Mustela putorius furo*	Y	Y	T
		*Mustela sibirica*	Y	N	T
Procyonidae	*Procyon*	*Potos flavus*	Y	Y	S
Felidae	*Felis*	*Felis catus*	Y	Y	T
	*Caracal*	*Caracal caracal*	Y	Y	T
	*Lynx*	*Lynx lynx*	Y	Y	T
	*Otocolobus*	*Otocolobus manul*	Y	Y	T
	*Puma*	*Puma concolor*	Y	Y	T
	*Panthera*	*Panthera leo*	Y	N	T
		*Panthera tigris tigris*	Y	Y	T
		*Panthera tigris altaica*	Y	Y	T
		*Panthera pardus orientalis*	Y	N	T
		*Panthera onca*	Y	N	T
		*Panthera uncia*	Y	N	T
	*Prionailurus*	*Prionailurus bengalensis*	Y	Y	T
Viverridae	*Paguma*	*Paguma larvata*	Y	Y	T
Herpestidae	*Suricata*	*Suricata suricatta*	Y	Y	T
Hyaenidae	*Hyaena*	*Hyaena hyaena*	Y	N	S

*Note*: Y, succeeded; N, failed; T, tissue; S, scat.

## References

[ece310135-bib-0001] Bensasson, D. , Zhang, D. X. , Hartl, D. L. , & Hewitt, G. M. (2001). Mitochondrial pseudogenes: evolution's misplaced witnesses. Trends in Ecology & Evolution, 16(6), 314–321. 11369110 10.1016/s0169-5347(01)02151-6

[ece310135-bib-0002] Bischof, R. , Gregersen, E. R. , Brøseth, H. , Ellegren, H. , & Flagstad, Ø. (2016). Noninvasive genetic sampling reveals intrasex territoriality in wolverines. Ecology and Evolution, 6(5), 1527–1536. 27087927 10.1002/ece3.1983 PMC4775525

[ece310135-bib-0003] Boyce, R. , Chilana, P. , & Rose, T. M. (2009). iCODEHOP: A new interactive program for designing COnsensus‐DEgenerate hybrid oligonucleotide primers from multiply aligned protein sequences. Nucleic Acids Research, 37(suppl_2), W222–W228. 19443442 10.1093/nar/gkp379 PMC2703993

[ece310135-bib-0004] Chaves, P. B. , Graeff, V. G. , Lion, M. B. , Oliveira, L. R. , & Eizirik, E. (2012). DNA barcoding meets molecular scatology: Short mtDNA sequences for standardized species assignment of carnivore noninvasive samples. Molecular Ecology Resources, 12(1), 18–35. 21883979 10.1111/j.1755-0998.2011.03056.x

[ece310135-bib-0005] De Barba, M. , Adams, J. R. , Goldberg, C. S. , Stansbury, C. R. , Arias, D. , Cisneros, R. , & Waits, L. P. (2014). Molecular species identification for multiple carnivores. Conservation Genetics Resources, 6, 821–824.

[ece310135-bib-0006] DeCandia, A. , Gaughran, S. , Caragiulo, A. , & Amato, G. (2016). A novel molecular method for noninvasive sex identification of order Carnivora. Conservation Genetics Resources, 8(2), 119–121.

[ece310135-bib-0007] Folmer, O. , Black, M. , Hoeh, W. , Lutz, R. , & Vrijenhoek, R. (1994). DNA primers for amplification of mitochondrial cytochrome c oxidase subunit I from diverse metazoan invertebrates. Molecular Marine Biology and Biotechnology, 3(5), 294–299. 7881515

[ece310135-bib-0008] Hebert, P. D. N. , Cywinska, A. , Ball, S. L. , & de Waard, J. R. (2003). Biological identifications through DNA barcodes. Proceedings of the Royal Society of London Series B: Biological Sciences, 270(1512), 313–321. 10.1098/rspb.2002.2218 PMC1691236 12614582

[ece310135-bib-0009] Huang, S. , Bininda‐Emonds, O. R. P. , Stephens, P. R. , Gittleman, J. L. , & Altizer, S. (2014). Phylogenetically related and ecologically similar carnivores harbour similar parasite assemblages. Journal of Animal Ecology, 83(3), 671–680. 24289314 10.1111/1365-2656.12160

[ece310135-bib-0010] Huber, S. , Bruns, U. , & Arnold, W. (2002). Sex determination of red deer using polymerase chain reaction of DNA from feces. Wildlife Society Bulletin, 30(1), 208–212.

[ece310135-bib-0011] Ibarguchi, G. , Friesen, V. L. , & Lougheed, S. C. (2006). Defeating numts: Semi‐pure mitochondrial DNA from eggs and simple purification methods for field‐collected wildlife tissues. Genome, 49(11), 1438–1450. 17426759 10.1139/g06-107

[ece310135-bib-0012] Ivanova, N. V. , Dewaard, J. R. , & Hebert, P. D. N. (2006). An inexpensive, automation‐friendly protocol for recovering high‐quality DNA. Molecular Ecology Notes, 6(4), 998–1002.

[ece310135-bib-0013] Kelly, M. J. , Betsch, J. , Wultsch, C. , Mesa, B. , & Mills, L. S. (2012). Noninvasive sampling for carnivores. In L. Boitani & R. A. Powell (Eds.), Carnivore Ecology and Conservation: A Handbook of Techniques (pp.47–69). Oxford University Press.

[ece310135-bib-0014] Kibbe, W. A. (2007). OligoCalc: an online oligonucleotide properties calculator. Nucleic Acids Research, 35(suppl_2), W43–W46. 17452344 10.1093/nar/gkm234 PMC1933198

[ece310135-bib-0015] Kim, J. H. , Antunes, A. , Luo, S. J. , Menninger, J. , Nash, W. G. , O'Brien, S. J. , & Johnson, W. E. (2006). Evolutionary analysis of a large mtDNA translocation (numt) into the nuclear genome of the *Panthera* genus species. Gene, 366(2), 292–302. 16380222 10.1016/j.gene.2005.08.023 PMC1592465

[ece310135-bib-0016] Kocher, A. , de Thoisy, B. , Catzeflis, F. , Huguin, M. , Valière, S. , Zinger, L. , Bañuls, A.‐L. , & Murienne, J. (2017). Evaluation of short mitochondrial metabarcodes for the identification of Amazonian mammals. Methods in Ecology and Evolution, 8(10), 1276–1283.

[ece310135-bib-0017] Korbie, D. J. , & Mattick, J. S. (2008). Touchdown PCR for increased specificity and sensitivity in PCR amplification. Nature Protocols, 3(9), 1452–1456. 18772872 10.1038/nprot.2008.133

[ece310135-bib-0018] Laguardia, A. , Wang, J. , Shi, F.‐L. , Shi, K. , & Riordan, P. (2015). Species identification refined by molecular scatology in a community of sympatric carnivores in Xinjiang, China. Zoological Research, 36(2), 72–78. 25855225 10.13918/j.issn.2095-8137.2015.2.72 PMC4790252

[ece310135-bib-0019] Lalitha, S. (2000). Primer premier 5. Biotech Software & Internet Report, 1(6), 270–272.

[ece310135-bib-0020] Larkin, M. A. , Blackshields, G. , Brown, N. P. , Chenna, R. , McGettigan, P. A. , McWilliam, H. , Valentin, F. , Wallace, I. M. , Wilm, A. , Lopez, R. , Thompson, J. D. , Gibson, T. J. , & Higgins, D. G. (2007). Clustal W and Clustal X version 2.0. Bioinformatics, 23(21), 2947–2948. 17846036 10.1093/bioinformatics/btm404

[ece310135-bib-0021] Li, J. , Cui, Y. , Jiang, J. , Yu, J. , Niu, L. , Deng, J. , Shen, F. , Zhang, L. , Yue, B. , & Li, J. (2017). Applying DNA barcoding to conservation practice: A case study of endangered birds and large mammals in China. Biodiversity and Conservation, 26(3), 653–668.

[ece310135-bib-0022] Lopez, J. V. , Yuhki, N. , Masuda, R. , Modi, W. , & O'Brien, S. J. (1994). Numt, a recent transfer and tandem amplification of mitochondrial DNA to the nuclear genome of the domestic cat. Journal of Molecular Evolution, 39(2), 174–190. 7932781 10.1007/BF00163806

[ece310135-bib-0023] Meusnier, I. , Singer, G. A. C. , Landry, J. F. , Hickey, D. A. , Hebert, P. D. N. , & Hajibabaei, M. (2008). A universal DNA mini‐barcode for biodiversity analysis. BMC Genomics, 9(1), 1–4. 18474098 10.1186/1471-2164-9-214 PMC2396642

[ece310135-bib-0024] Michalski, F. , Valdez, F. P. , Norris, D. , Zieminski, C. , Kashivakura, C. K. , Trinca, C. S. , Smith, H. B. , Vynne, C. , Wasser, S. K. , Metzger, J. P. , & Eizirik, E. (2011). Successful carnivore identification with faecal DNA across a fragmented Amazonian landscape. Molecular Ecology Resources, 11(5), 862–871. 21676206 10.1111/j.1755-0998.2011.03031.x

[ece310135-bib-0025] Modi, S. , Mondol, S. , Ghaskadbi, P. , Hussain, Z. , Nigam, P. , & Habib, B. (2018). Noninvasive DNA‐based species and sex identification of Asiatic wild dog. Journal of Genetics, 97(5), 1457–1461. 30555094

[ece310135-bib-0026] Mumma, M. A. , Zieminski, C. , Fuller, T. K. , Mahoney, S. P. , & Waits, L. P. (2015). Evaluating noninvasive genetic sampling techniques to estimate large carnivore abundance. Molecular Ecology Resources, 15(5), 1133–1144. 25693632 10.1111/1755-0998.12390

[ece310135-bib-0027] Pietsch, S. J. (2012). DNA barcoding & multi‐isotopic fingerprinting: A novel forensic toolbox for the rapid identification of illegal trade in endangered wildlife species. Universitäts‐und Landesbibliothek Bonn.

[ece310135-bib-0028] Rodgers, T. W. , & Janečka, J. E. (2013). Applications and techniques for non‐invasive faecal genetics research in felid conservation. European Journal of Wildlife Research, 59(1), 1–16.

[ece310135-bib-0029] Rodriguez‐Castro, K. G. , Saranholi, B. H. , Bataglia, L. , Blanck, D. V. , & Galetti, P. M. (2020). Molecular species identification of scat samples of south American felids and canids. Conservation Genetics. Resources, 12(1), 61–66.

[ece310135-bib-0030] Rose, T. M. , Henikoff, J. G. , & Henikoff, S. (2003). CODEHOP (COnsensus‐DEgenerate hybrid oligonucleotide primer) PCR primer design. Nucleic Acids Research, 31(13), 3763–3766. 12824413 10.1093/nar/gkg524 PMC168931

[ece310135-bib-0031] Rose, T. M. , Schultz, E. R. , Henikoff, J. G. , Pietrokovski, S. , McCallum, C. M. , & Henikoff, S. (1998). Consensus‐degenerate hybrid oligonucleotide primers for amplification of distantly related sequences. Nucleic Acids Research, 26(7), 1628–1635. 9512532 10.1093/nar/26.7.1628 PMC147464

[ece310135-bib-0032] Santini, A. , Lucchini, V. , Fabbri, E. , & Randi, E. (2007). Ageing and environmental factors affect PCR success in wolf (*Canis lupus*) excremental DNA samples. Molecular Ecology Notes, 7(6), 955–961.

[ece310135-bib-0033] Schmidt, J. H. , Rattenbury, K. L. , Robison, H. L. , Gorn, T. S. , & Shults, B. S. (2017). Using non‐invasive mark‐resight and sign occupancy surveys to monitor low‐density brown bear populations across large landscapes. Biological Conservation, 207, 47–54.

[ece310135-bib-0034] Schrader, C. , Schielke, A. , Ellerbroek, L. , & Johne, R. (2012). PCR inhibitors–occurrence, properties and removal. Journal of Applied Microbiology, 113(5), 1014–1026. 22747964 10.1111/j.1365-2672.2012.05384.x

[ece310135-bib-0035] Schwartz, M. K. , & Monfort, S. L. (2008). Genetic and endocrine tools for carnivore surveys. In R. Long , P. MacKay , J. Ray , & W. Zielinski (Eds.), Noninvasive survey methods for north American carnivores (pp. 228–250). Island Press.

[ece310135-bib-0036] Smart, U. , Budowle, B. , Ambers, A. , Soares Moura‐Neto, R. , Silva, R. , & Woerner, A. E. (2019). A novel phylogenetic approach for de novo discovery of putative nuclear mitochondrial (pNumt) haplotypes. Forensic Science International: Genetics, 43, 102146. 31446343 10.1016/j.fsigen.2019.102146

[ece310135-bib-0037] Song, H. , Buhay, J. E. , Whiting, M. F. , & Crandall, K. A. (2008). Many species in one: DNA barcoding overestimates the number of species when nuclear mitochondrial pseudogenes are coamplified. Proceedings of the National Academy of Sciences of United States of America, 105(36), 13486–13491. 10.1073/pnas.0803076105 PMC2527351 18757756

[ece310135-bib-0038] Swindell, S. R. , & Plasterer, T. N. (1997). Sequence Data Analysis Guidebook (pp. 75–89). Springer.

[ece310135-bib-0039] Symondson, W. O. C. (2002). Molecular identification of prey in predator diets. Molecular Ecology, 11(4), 627–641. 11972753 10.1046/j.1365-294x.2002.01471.x

[ece310135-bib-0040] Taberlet, P. , Waits, L. P. , & Luikart, G. (1999). Noninvasive genetic sampling: Look before you leap. Trends in Ecology & Evolution, 14(8), 323–327. 10407432 10.1016/s0169-5347(99)01637-7

[ece310135-bib-0041] Tu, S. L. , Staheli, J. P. , McClay, C. , McLeod, K. , Rose, T. M. , & Upton, C. (2018). Base‐by‐base version 3: New comparative tools for large virus genomes. Viruses, 10(11), 637. 30445717 10.3390/v10110637 PMC6265842

[ece310135-bib-0042] Verscheure, S. , Backeljau, T. , & Desmyter, S. (2015). In silico discovery of a nearly complete mitochondrial genome Numt in the dog (*Canis lupus familiaris*) nuclear genome. Genetica, 143(4), 453–458. 25991039 10.1007/s10709-015-9844-3

[ece310135-bib-0043] Wang, D. , Hu, Y. , Ma, T. , Nie, Y. , Xie, Y. , & Wei, F. (2016). Noninvasive genetics provides insights into the population size and genetic diversity of an Amur tiger population in China. Integrative Zoology, 11(1), 16–24. 26663614 10.1111/1749-4877.12176

[ece310135-bib-0044] Ward, R. D. , Zemlak, T. S. , Innes, B. H. , Last, P. R. , & Hebert, P. D. N. (2005). DNA barcoding Australia's fish species. Philosophical Transactions of the Royal Society B: Biological Sciences, 360(1462), 1847–1857. 10.1098/rstb.2005.1716 PMC1609232 16214743

[ece310135-bib-0045] Wei, F. W. , Hu, Y. B. , Zhu, L. F. , Bruford, M. W. , Zhan, X. J. , & Zhang, L. (2012). Black and white and read all over: The past, present and future of giant panda genetics. Molecular Ecology, 21, 5660–5674. 23130639 10.1111/mec.12096

[ece310135-bib-0046] Woerner, A. E. , Cihlar, J. C. , Smart, U. , & Budowle, B. (2020). Numt identification and removal with RtN! Bioinformatics, 36(20), 5115–5116. 32706871 10.1093/bioinformatics/btaa642

[ece310135-bib-0047] Yu, L. , & Zhang, Y. P. (2006). Summary of phylogeny in mammalian order Carnivora. Zoological Research, 27(6), 657–665.

[ece310135-bib-0048] Zemanova, M. A. (2021). Noninvasive genetic assessment is an effective wildlife research tool when compared with other approaches. Genes, 12(11), 1672. 34828277 10.3390/genes12111672 PMC8625682

[ece310135-bib-0049] Zhang, D. X. , & Hewitt, G. M. (1996). Nuclear integrations: Challenges for mitochondrial DNA markers. Trends in Ecology & Evolution, 11(6), 247–251. 21237827 10.1016/0169-5347(96)10031-8

